# Churg-Straus syndrome: A case report

**Published:** 2017-07-06

**Authors:** Safa Najmi, Armaghan Ghareaghaji-Zare, Saeed Ghazanfari-Amlashi

**Affiliations:** 1 Department of Neurology, School of Medicine, St. Louis University, St. Louis, USA; 2 Department of Dermatology, School of Medicine, Tabriz University of Medical Sciences, Tabriz, Iran; 3 Department of Neurology, School of Medicine, Kurdistan University of Medical Sciences, Sanandaj, Iran

**Keywords:** Churg-Strauss Syndrome, Eosinophilia, Cerebral Infarction, Anticoagulants

Churg-Strauss syndrome is a rare autoimmune disorder characterized by excess circulating, tissue eosinophils, and vasculitis, which affects the lung and skin. The syndrome occurs in patients with a history of asthma or allergy.^1^ Neuropathy develops in approximately 3/4 of the patients usually as mononeuritis multiplex. Centrally accentuated antineutrophil cytoplasmic antibody (CANCA) is generally found in more than half of the cases, while central nervous system (CNS) manifestations are relatively unusual and include headache, convulsion, hemiplegia, and brainstem signs.^2^

We report here a 42-year-old man with a history of severe asthma and rhinitis in the past 4 years prior to the first admission. The patient was presented with difficulty in walking and weakness of lower limbs. In addition, he had a history of flu vaccination about 1.5 months before his neurological symptoms. The patient also had a history of skin lesions of hemorrhagic bulla and palpable purpura few days after injection on the lower limbs. At that time, a dermatologist visited the patient and a biopsy was taken with the impression of vasculitis. Eosinophilic dermal infiltration and leukocytoclastic vasculitis (LCV) were demonstrated in the biopsy specimen. 

Findings on the first day of admission were as follows: electromyogram/nerve conduction study (EMG/NCS) were compatible with subacute mixed type (demyelination and axonal) of inflammatory polyradiculopathy [Guillain-Barré syndrome (GBS)]. At the complete blood cell (CBC) exam, white blood cells (WBCs) were 14.21 (10^3^/µl) with eosinophilia (40%) (Table 1).

Erythrocyte sedimentation rate (ESR) was 43 and 84 at 1^st^ and 2^nd^ hours, respectively; C-reactive protein (CRP) was negative, and the rheumatoid factor (RF) was ++. In addition, antinuclear antibodies (ANA), anti-cyclic citrullinated peptide (anti-CCP) antibody, cytoplasmic-antineutrophil cytoplasmic antibody (c-ANCA), and perinuclear-anti-neutrophil cytoplasmic antibody (p-ANCA), and anti-phospholipid antibody were found to be negative. Lipid profiles and liver function tests were in the normal range. Brain and thoracolumbar spine magnetic resonance imaging (MRI) results were not significant, except for increased mucosal thickening at both maxillary and sphenoidal sinuses.

**Table 1:** Complete blood cell (CBC) results

**Variable**	**Results**	**Normal range**	**Unit**
WBC	14.21*	4.8-10.8	10^3^/µl
RBC	4.96	4.20-6.10	10^3^/µl
HGB	13.6	12.0-18.0	g/dl
HCT	39.8	37.0-52.0	%
MCV	80.4	79.0-99.0	femtoliters/cell
MCH	27.5	26.0-32.0	pg/cell
MCHC	34.2	31.5-36.0	g/dl
PCT	0.34	0.12-0.36	%
RDW	14.7	11.5-15.0	%
HDW	2.88	2.20-4.00	g/dl
PLT	459*	130-400	10^3^/µl
MPV	7.5	6.4-11.1	femtoliters/cell
PDW	66.1	25.0-75.0	%
NEUT (%)	36.9*	40.0-74.0	%
LYMPH (%)	16.7*	19.0-48.0	%
MONO (%)	4.9	3.4-9.0	%
EOS (%)	40*	0-7	%
BASO (%)	0.3	0.0-1.5	%
LUC (%)	1.3	0.0-4.0	%
NRBC (%)	0	0.0-2.0	%
NEUT	5.24	1.90-8.00	10^3^/µl
LYMPH	2.37	1.90-5.20	10^3^/µl
MONO	0.69	0.16-1.00	10^3^/µl
EOS	5.68*	0.00-0.80	10^3^/µl
BASO	0.05	0.00-0.20	10^3^/µl
LUC	0.19	0.00-0.40	10^3^/µl

WBC: White blood cell; RBC: Red blood cell; HGB: Hemoglobin; HCT: Hematocrit; MCV: Mean corpuscular volume; MCH: Mean corpuscular hemoglobin; MCHC: Mean corpuscular hemoglobin concentration; PCT: Prolactin count; RDW: Red blood cell distribution width; HDW: Hemoglobin distribution width; PLT: Platelet; MPV: Mean platelet volume; PDW: Platelet distribution width; NEUT: Neutrophil; LYMPH: lymphocytes; MONO: Monocyte; EOS: Eosinophil; BASO: Basophil; LUC: Large unstained cell; NRBC: Nucleated red blood cell

* ABN Scattergram

In this admission, intravenous immunoglobulin (IVIG) was administered with the impression of GBS. There was a little improvement but other signs and symptoms appeared progressively in the following year; the symptoms included limb tremor, numbness of fingers, dysarthria, dysphagia, gait impairment, arthralgia, and incontinency.

After 11 months, the patient was admitted again with unilateral sudden visual loss. Clinical examination, according to our last visit, showed bilateral wheezing and fever (38.3 ºC oral). Blood pressure (BP), pulse rate (PR), and respiratory rate (RR) were in normal range. Skin was warm and moist, but no specific lesion was detected. On neurologic examination, there was no response to direct light in the right eye; however, the indirect exam was normal, and there was evidence of central retinal artery occlusion (CRAO) in the right eye, viewed with an ophthalmoscope. Right nasolabial fold was flattened. Muscle force of lower limbs was 4/5, while the upper limbs were normal.

The deep tendon reflex of upper limbs was +++ with obvious rigidity, and the plantar reflex was equivocal. The peripheral blood examination disclosed leukocytosis (WBC: 18.47 10^3^/µl) with eosinophilia (23.6%); additionally, the C4 level was low (0.127 g/l with reference range of 0.165-0.380) but C3 and CH50 levels were in the normal range. p-ANCA, c-ANCA, ANA, and anti-double stranded DNA (anti-dsDNA) antibodies were tested again, and found to be negative. Other findings were as: CRP: +++, ESR at 1 hour: 122, RF: ++, prothrombin time (PT): 12.7 s, partial thromboplastin time (PTT): 28.0 s, and international normalized ratio (INR): 1.1.

Cardiac echocardiogram showed ejection fraction of 40-45% as well as anteroseptal hyperkinesia. Both carotids and vertebral arteries were normal in the cervical duplex study. Spiral computed tomography (CT) scan of chest revealed pneumonia and effusion with pleural thickening. Brain MRI findings disclosed diffused symmetrically increased signal intensity at the hemispheric white matter, pons, internal capsule, cerebellar hemispheres, and peduncles bilaterally, showing restricted diffusion on diffusion-weighted images. Mucosal thickening was observed in the maxillary, ethmoidal, sphenoidal, and frontal sinuses (pansinusitis) (Figure 1).

**Figure 1:**
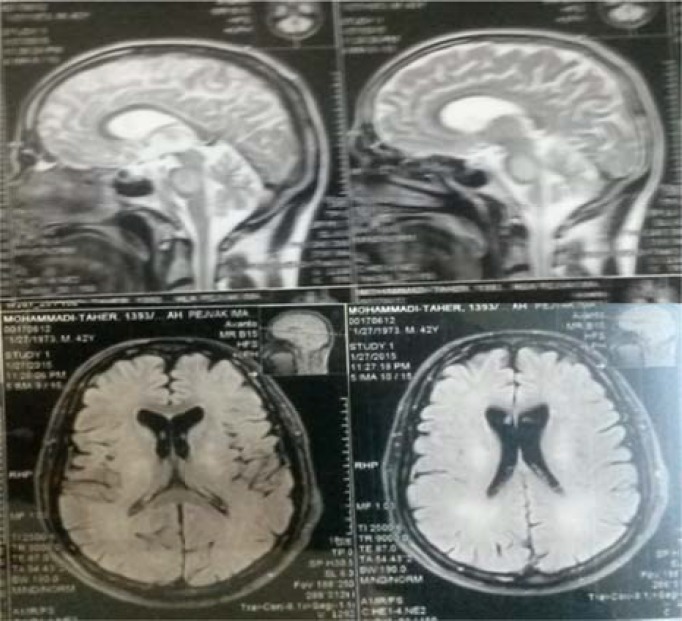
Mucosal thickening in the maxillary, ethmoidal, sphenoidal, and frontal sinuses

The presence of all these findings led to the diagnosis of Churg-Strauss syndrome by American College of Rheumatology (ACR) criteria. The patient was treated with prednisolone (1 mg/kg/day) and cyclophosphamide (150 mg/day). After a month, he came back to the hospital with the complaint of right lower limb pain. This time, we made a decision to keep the patient on warfarin, in addition to previous drugs, and waited to see what would happen later.

Recent studies demonstrated that neurologic manifestations are very interesting in these patients.^2^^,^^3^ Neurologic manifestations, in a variety of reports, include subarachnoid hemorrhage, intracerebral hemorrhage, cerebral/cerebellar infarct or gliosis, and spinal cord lesion.^4^ MRI performed in the first admission showed no abnormalities; nevertheless, there was evidence of peripheral nervous system (PNS) involvement, as misdiagnosed by GBS. However, in the second admission, the brain MRI revealed many hypersingnal foci for vasculitis involvement of the brain parenchyma; indeed, the patient had cerebral/cerebellar infarcts.

The fundamental question that arises here is: what is the possible explanation for these lesions in the brain? It can be due to cardiac embolism, vasculitis, or hypercoagulation. In our patient, we found no cardiac embolism source for his brain lesions, and vasculitis and hypercoagulation remained blameful for CNS manifestation.

He was treated with prednisone (1 mg/kg/day) and cyclophosphamide (150 mg/day), as a usual treatment and standard initial therapy.^5^ His signs and symptoms were improved significantly, but the patient experienced a severe attack in less than one month of the therapy, which led to amputation of his leg beneath the knee. At that time, we found out that he may need something more than the usual and standard treatment; therefore, we decided to add warfarin therapy with checking the INR within 2-3 weeks. We followed the patient for about 5 months, and found that he did not experience new attacks after warfarin therapy. Further studies are required to confirm effectiveness of anticoagulation therapy for severe cases of Churg-Strauss syndrome.

## Notes:

**How to cite this article:** Najmi S, Ghareaghaji-Zare A, Ghazanfari-Amlashi S. Churg-Straus Syndrome: A Case report. Iran J Neurol 2017; 16(3): 159-61.

## References

[1] Grau RG (2008). Churg-Strauss syndrome: 2005-2008 update. Curr Rheumatol Rep.

[2] Ropper A, Samuels M, Klein J (2014). Adams and Victor's principles of neurology.

[3] Wolf J, Bergner R, Mutallib S, Buggle F, Grau AJ (2010). Neurologic complications of Churg-Strauss syndrome--a prospective monocentric study. Eur J Neurol.

[4] Cheng MJ, Huang PH, Liao PW, Chen JT, Chiang TR (2012). Multiple cerebral and cerebellar infarcts as the first clinical manifestation in a patient with Churg-Strauss syndrome: case report and literature review. Acta Neurol Taiwan.

[5] Bosch X, Guilabert A, Espinosa G, Mirapeix E (2007). Treatment of antineutrophil cytoplasmic antibody associated vasculitis: a systematic review. JAMA.

